# Huntington's Disease: A Clinical Review

**DOI:** 10.7759/cureus.28484

**Published:** 2022-08-27

**Authors:** Rajeshwar Andhale, Deepti Shrivastava

**Affiliations:** 1 Medicine, Jawaharlal Nehru Medical College, Datta Meghe Institute of Medical Science, Wardha, IND; 2 Department of Obstetrics and Gynecology, Jawaharlal Nehru Medical College, Datta Meghe Institute of Medical Science, Wardha, IND

**Keywords:** genetic disorder, autosomal dominant disease, chorea, cag repeat, huntington's disease

## Abstract

The Huntington's gene on chromosome 4 has a dominantly inherited CAG trinucleotide repeat expansion, ultimately resulting in Huntington's disease (HD), a completely penetrant neurological condition. The frequency is 10-100 times higher in the population descended from Europe than in East Asia. Through various processes, including impairment of proteostasis, transcription, and cell function, as well as direct toxicity of the mutant protein, mutated huntingtin triggers neuronal malfunction and loss at the cellular level. As the disease worsens, the brain becomes affected together with the striatum's initial macroscopic alterations. Since there are presently few medications that can change the course of the disease, palliative therapy, and symptom control are the cornerstone of treatment. Studying the cellular pathology and gross structural changes to the brain which occur as the illness advances have made enormous progress in recent years. There's been a substantial increase in medical studies and possible treatment options over the past ten years. The new treatments that aim to reduce amounts of mutant huntingtin are the most optimistic. However, one strategy is antisense oligonucleotide treatment, for which clinical trials are currently being conducted. These control trials might help us get another inch ahead of managing and perhaps even eliminating this nasty disease.

## Introduction and background

Around 1842, Waters reported the first account of a person who had what is now recognized as Huntington's chorea. However, it wasn't until 1872, following George Huntington's presentation and explanation of the condition, that it was awarded the title of Huntington's chorea. It is a neurological condition that develops in mid-life and is passed down within households from successive generations. It is marked by forgetfulness, psychosocial problems, and involuntary choreatic gestures [1]. Its title stayed unchanged for many years until the 1990s, when Huntington's disorder was officially defined after substantial non-motor complaints. The Huntington's disease (HD) gene was identified in 1993, just after correlation off chromosome 4 was placed in 1983 [2]. There had been a dramatic rise in attention to HD and other neurocognitive illnesses around that time. Nevertheless, due to its single-gene origin and complete penetrance, HD is among the most curable neurodegenerative disorders. Thanks to the development of novel therapeutic strategies which can specifically target the Huntingtin gene and inhibit the synthesis of the damaging mutant huntingtin protein over the past ten years [3]. A solid reason for how to treat this terrible disease emerged for the first time when the gene was discovered, providing new research avenues and modeling possibilities. There are already several symptomatic therapies obtainable. However, improved modification medications are still required.

## Review

Etiology 

A CAG trinucleotide repeat increase in the huntingtin gene on chromosome 4 results in Huntington's disease, which is an autosomal dominantly inherited condition. An aberrant huntingtin protein with an extended polyglutamine sequence is formed as a result [2]. A person is guaranteed to get the disease if they have more than 39 CAG repeats, but penetrance is minimized among 36 and 39 repetitions. A parent with a CAG repeat length in the middle of the range could have a child with an increased pathogenic repeat length, which is an example of expectation whenever the gene is handed down to the father's side. It's attributable to the fact that sperm cells exhibit more repeat variability and bigger repeat sizes compared to tissues throughout the body [4].

Epidemiology

In European communities, the prevalence of Huntington's disease ranges from 10 to 13 per 100,000 people. With a frequency of 1-7 per million, Huntington's disease is substantially less frequent in East Asia. Black inhabitants in South Africa have lower rates than white and mixed communities. The genetic variations in the huntingtin (HTT) gene are attributable to differences in illness frequency among ethnic communities. Typical CAG repeat length is longer in communities having increasing incidence levels [5].

Molecular pathogenesis

**Figure 1:**
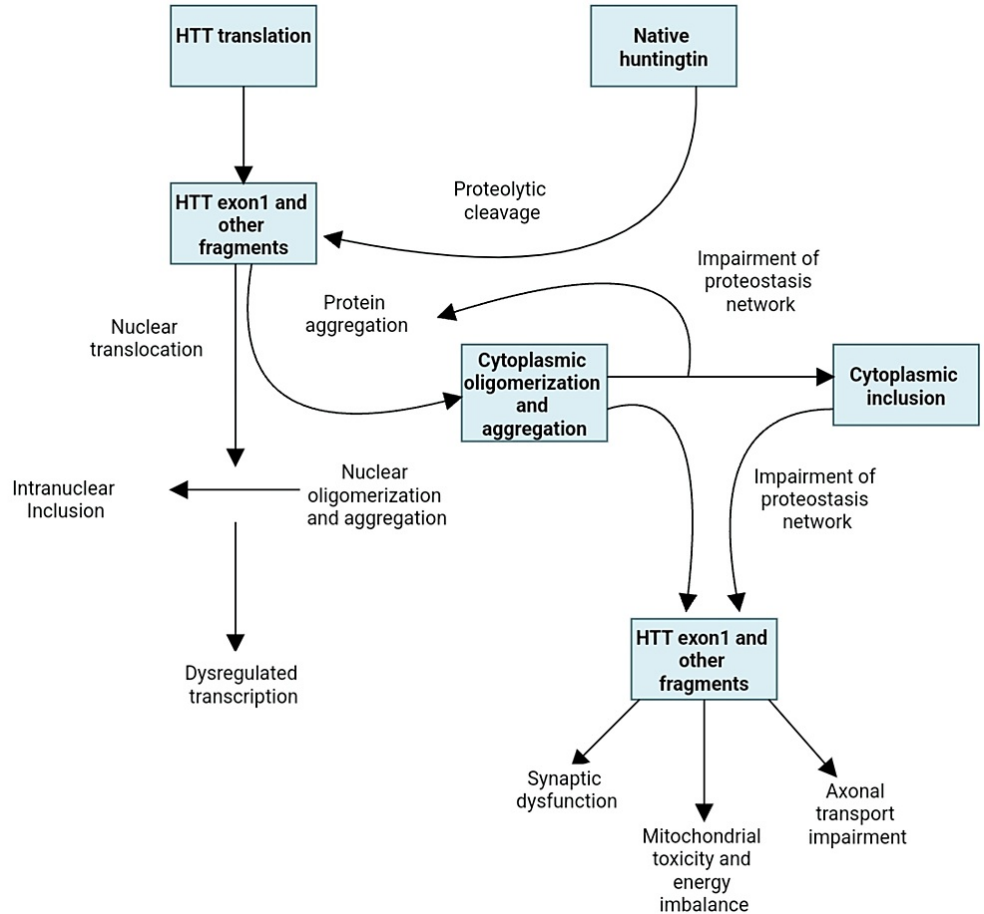
Huntington's disease pathogenetic cellular mechanisms HTT - huntingtin

Numerous pathways are involved in the malfunction and neuronal death caused by the mutated huntingtin gene. The direct impacts of the locus one mutant huntingtin (mHTT) segment, the tendency of mHTT to produce aberrant aggregates as well as its implications on cellular homeostasis, neuronal transport, transcription, translation, mitochondrial function, and synaptic function [5,6] (Figure 1). The striatal medium spiny neurons are particularly susceptible to the adverse effects of that. Striatal pathology progresses in two phases. The first phase is marked by the loss of medium spiny neurons (MSNs) of the indirect pathway, which causes a hyperkinetic phenotype, and the second phase is characterized by the loss of medium spiny neurons of the direct path, which causes a hypokinetic trait [7]. Unknown factors may have contributed to the indirect pathway of MSNs selective susceptibility; however, D2 dopamine receptors may play a role because they have been linked to Huntington's pathogenesis and are expressed indirectly but not directly MSNs [8]. Other speculations also include depletion of brain-derived neurotrophic components, glutamate excitotoxicity from cortico-striatal estimates, and the detrimental consequences of repeat-associated non-ATG translation proteins [6,9].

Macroscopic pathology

The caudate and putamen exhibit extensive shrinkage and medio-lateral, dorso-ventral, and audio-rostral gradient of degradation, which has been observed in postmortem investigations, and even to a lesser extent, the nucleus accumbens and globus pallidus are also impacted [10]. There are five grades in the pathology of Huntington's disease (Table 1), according to a defined classification system [11].

**Table 1:** Grading of Huntington's disease [11]

	Clinical evidence	Macroscopic anomalies	Microscopic anomalies
Grade 0	Present	No	No
Grade 1	+	No	Moderate fibrillary astrocytosis
Grade 2	+	+ caudate/putamen; - globus pallidus	+
Grade 3	+	The lateral segment of globus pallidus (fibrillary astrocytosis)	+
Grade 4	+	Shrunken caudate and small nucleus accumbens	+

Clinical narration

The following are the pathognomic indications and symptoms of Huntington's disease: intellectual, musculoskeletal, and psychiatric disturbances. Unintentional weight reduction, sleep and body clock disruptions, and disruption of the autonomic nervous system are additional, relatively poorly known, yet frequent and frequently incapacitating Huntington's disease symptoms. With such a range of 2-80 years, its average age at onset is somewhere between 35-45 years. The condition usually lasts 16-18 years. As the condition advances, increased dependence on everyday tasks and eventually leads to loss of life. Lung infections are the leading cause of mortality; second is suicide. Neuromuscular manifestations: Involuntary, undesired motions are the hallmark of motor impairments. Usually, the motions frequently involve minor face muscles and also distal extremities like toes and fingers. These muscle spasms are often unnoticeable to onlookers or could be attributed to anxiety. In an ordinary routine, a person's gait can become erratic, and they may appear to be a little inebriated. These undesirable movements spread widely to other groups of muscles, from the caudal towards the proximal part. All of the patient's awake time is spent with choreatic gestures. Although there isn't a single pattern, facial choreography may lead to facial muscle contractions, such as an eyebrow raise, blinking, head bowing or turning, or tongue sticking out with pouting lips. The lengthy back muscles' extension motions are most noticeable. In specific individuals, speaking and swallowing gradually grow more challenging, making choking possible at any time. Eventually, the patient ultimately develops muteness. Difficulty in speech and difficulty in eating become increasingly noticeable as the illness progresses. These individuals then develop slowness of movement and difficulty in starting the movement. Each person has a different level of hypokinesia and chorea. The two extremes are the juvenile patient with overbearing stiffness and the very elderly patient whose been ill for quite a long time and is also seriously affected in the final phase of the illness, bed bound with stiffness and flexion contractures in the limbs. Sluggish movements and higher muscle tone, which cause aberrant alignments like torticollis as well as rotation of the trunk or extremities, are hallmarks of dystonia. The initial motor indication of Huntington's illness may be dystonia. Tics are another type of undesired movement, though they are relatively uncommon. Cerebellar complaints may manifest erratically, much the same as hypo- and hypermetria. In many instances, movement is compared to being "inebriated" or experiencing cerebellar ataxia. It can be very challenging to distinguish between choreatic and ataxic gait. Pyramidal signs are unintentionally present.

With duration, the neurological disturbance has a growing impact on daily tasks. Additionally, it gets more and more challenging to perform essential everyday functions, including getting out of bed, having a bath, clothing, using the restroom, house cleaning, cooking, and dining. Motor symptoms will ultimately hinder the effectiveness, depending on what type of activity the patient conducts, even if psychological and intellectual alterations are still in the foreground. 

Quite commonly and often before motor symptoms even appear, psychological symptoms are seen in the early phase of the disease. Pertaining to the research technique, the proportion of patients exhibiting psychological symptoms ranges from 30 to 77 percent [12]. This leads to a very detrimental influence on the productivity of families due to daily life disturbances [13]. The fact that Huntington's disease also causes weight reduction and apathy, so diagnosis is challenging. Typically, there are emotions of guilt, fear, and low self-confidence. Only apathy but not anxiety or depression is correlated with the disease phase. Early symptomatic individuals and pre-manifest gene carriers are more likely to commit suicide. The most dangerous times for suicide occur around the time of gene testing, as well as the phase when individuality starts to wane. Nervousness additionally occurs 30-60% of the time, usually in connection with ambiguity over the onset and progression of the condition. The patient's life may be disrupted by obsessive-compulsive disorder, which can also cause anger and violence. In hindsight, impatience is frequently the first indication though it actually manifests at all phases of the illness [12]. There are many different ways to exhibit impatience, ranging from heated arguments to actual assault. The apathy condition is said to include a decline in enthusiasm as well as a rise in passive conduct. Differentiating between depression and disinterest can be challenging. Usually, in the final phase of the disease, psychosis can develop. This typically occurs in conjunction with mental deterioration. The entire clinical profile resembles delusional and auditory delusions in schizophrenia. Hypersexuality can seriously complicate a marriage in its initial phases but is later characterized by hyposexuality. The other primary Huntington's symptom is mental deterioration, which can be seen before the initial motor symptoms and can also be relatively minor in the early phases of the disease till the disease advances. Executive functions are notably affected by mental alterations. Cognitive and physical activity are goal-directed and designed under typical circumstances. Individuals having Huntington's disease are unable to discern between what is essential and what can be neglected in everyday life. Individuals who once had no trouble planning or managing their lives are now unable to do so. Individuals become less mentally versatile and are unable to adapt their thinking. Individuals no longer respond as they did in the past or as the surroundings would anticipate, which hampers problems. Speech is comparatively unaffected. Albeit the semantic recall can be partially preserved, recollection ultimately deteriorates over time. All cognitive functions experience substantial retardation [14].

Additional signs and symptoms are unintentional weight reduction has now been documented in all individuals from the beginning. With increased awareness of this issue, weight loss appears to be less severe due to its many origins. Even though it would seem reasonable to assume that chorea would be the primary factor in losing weight, the research has found that neither chorea nor any other movement disorders are linked to weight reduction. There is a correlation between the CAG repeat length and other factors [15]. Logistical challenges, including sluggishness, diminished appetite, trouble managing food, and swallowing difficulties, undoubtedly play a part. Destruction of hypothalamic neurons is a contributing cause as well [16,17].

HD patients' sleep and body clock abnormalities have only lately received attention [18]. Episodes of excessive sweating might be brought on by autonomic abnormalities. Juvenile HD is the name of the condition if the first clinical manifestations appear before 20 years of age. The clinical images are often social abnormalities and academic failure. Musculoskeletal activity often exhibits dystonic and hypokinetic features. Chorea usually emerges in the second decade and is hardly seen in the first. Epileptic seizures occur often. For most instances, the CAG repeat length is greater than 53. Usually, a father is a parent who is impacted in 75% of cases involving children [19].

Evaluation

For the individual, household, and caretakers, the clinical evaluation of Huntington's manifestations is crucial. Numerous scales have already been established in order to monitor the individual methodically, primarily for study purposes. The Unified Huntington Disease Rating Scale (UHDRS) scale and the Shoulson and Fahn capabilities scale are the most well-known [20-22]. There are several other scales still being used, such as one for liveability. Over six thousand patients across western countries are currently using a comprehensive set of assessment measures developed by the European Network for Huntington's disease (EHDN) [23].

Diagnostics

The commencement of motor impairment according to the Unified Huntington's disease Rating Scale, total motor score, and a diagnostic confidence score is required for the confirmation of Huntington's disease, as makes a proven family history or even a positive genetic test. A score of four indicates motor onset or "obvious" Huntington's disease. This ranges from zero to four. However, the premanifest phase of the illness, which can be detected up to 11-16 years before the onset of manifest disease, refers to mild motor, intellectual and psychological deficits [24].

Differential diagnoses

A Huntington's phenocopy is the term for the chorea, neurocognitive, and psychiatric disorder trio in the absence of an mHTT variant. Several genetic disorders might manifest as Huntington's phenocopies, even though confirmation could only be made in about 2-4% [25] of these cases. The two that are most prevalent in western populations are spinocerebellar ataxia 17 [25] and C9orf72 [26]. Particular attention should be paid to dentatorubral-pallidoluysian atrophy in the event of convulsions. Neuroferritinopathy and neurodegeneration with brain iron accumulation are two iron deposition illnesses that can show up on atypical diagnostic imaging. On peripheral smear of blood, aberrant acanthocytes can be observed in the case of neuroacanthocytosis. The most frequent cause of Huntington's phenocopies among Nigro people is Huntington's disease-like syndrome 2, according to studies [26,27].

Genetic guiding

Linking analysis was used for the first time to enable premanifest identification after the gene's chromosome 4 location was discovered in early 1983 [28]. Findings from the linkage studies were given to the candidate with a confidence of 93% at first and a probability of roughly 98% subsequently. Genuine premanifest diagnosis might be given to persons at risk of Hungtington once the CAG repetition on chromosome 4 was identified in early 1993. It served as a model for how to handle brand-new concerns and problems as it was the first illness for which this treatment got genuinely attainable. The Hungtington society, which included researchers, medical professionals, and laypeople, wrote a manifesto [29]. The foregoing was the accepted practice (Table 2).

**Table 2:** Steps of genetic guiding in Huntington's disease

Step	Guidelines
Step 1	Clinical genetics consultation, ideally in conjunction with a psychiatrist and a neurologist.
Step 2	A second session that includes a blood sample is held four to six weeks later.
Step 3	There will be a session with disclosure following 6-8 weeks.

The protocol was expanded to include the need for the candidate to make every attempt to obtain a result from the parent who has a 50% chance of having Huntington's disease. Eventually, the candidate who is 25% in danger can take an assessment [30].

Prenatal diagnosis

Prenatal detection is also feasible since the test may be carried out on every cell that has nuclei that contain the genetic code. Chorionic villus testing can be done around weeks 10-12, and amniocentesis and genetic testing can also be done around weeks 15-17 of gestation. To avoid unintended revelation for two people at once, the process is only started if, indeed, the parents are aware of their very own genetic condition. If somehow the Huntington gene is discovered in the fetus, the technique will be used to terminate the pregnancy. The parents can not be coerced into accepting this judgment. By correlating the genetic profile of the fetus with the grandparents, one might choose an exclusion test if somehow the parents have still not had their genotypes determined. In this case, the outcome seems to be either 0% danger for the embryo, in whose case the parent retains their 50% status, or perhaps 50% danger for the embryo. Although the embryo does have a chromosome from the grandparent who has the disorder, this is unknown to which chromosome the Hungtington gene is linked. In this scenario, the danger to the embryo is equal to that of the parents, and indeed, the couples have the option of aborting a baby who is 50% in danger. Pre-implantation diagnosis is also being made available in a number of nations during the past ten years. The assisted reproduction process is the first step. Single-cell from the eight-cell stage of the embryo is taken out for genetic analysis. To enable a typical gestation to progress, the embryo lacking the extended CAG repetition is implanted in the womb of the mother. The chromosomal makeup of the father and mother should be established before beginning this treatment, albeit not all nations adopt this philosophy [31].

Management

Regardless of the fact that the pathophysiology of Huntington's is still unknown and there is no known treatment, there are several treatment alternatives for managing the disease's manifestations and indicators with the goal of improving the quality of care. It is often not required to treat the symptoms, despite the fact that many of them might be. The patient's functional capabilities dictate whether or not the medication is needed. There is indeed a dearth of data on the prescription or recommended dose for any clinical symptoms. Consequently, pharmacological intervention is tailored to the person and is dependent on professional judgment. Both prescription drug use, as well as non-drug counseling, are part of the treatment. Surgery isn't really a significant factor in Hungtington and will only be briefly discussed.

Neurocognitive Symptoms

One of the Hungtington symptoms that manifests more visibly immediately is chorea. Tetrabenazine seems to be the only medication approved, primarily to manage chorea [32]. At dosages between 50 to 75 milligrams each day, this blocker of synaptic vesicular amine transport offers a long-lasting antichoreic action. Insomnia, stress, anxiousness, and unrest are among the adverse effects [33]. Deutetrabenazine is a deuterium-containing variant of tetrabenazine. This causes a longer half-life and reduced variability during metabolism. Deutetrabenzine dramatically reduces chorea when, especially in comparison to a placebo, according to the first Hunigtion study [34]. Although tetrabenazine and deutetrabenzine have not yet been compared directly in studies, there seems to be some evidence that deutetrabenzine may have lesser side effects, such as anxiety and insomnia [35].

In a randomized study, the neuroleptic sulpiride proved effective in the management of chorea. Additional neuroleptics, such as risperidone and quetiapine, are also often used in clinical settings, drowsiness and excess weight gain being their main prevalent adverse effects. Physiotherapy is frequently used to address additional motor complaints, such as strange gait, poor balance, and recurrent drops [33]. Psychological symptoms' recommendations regarding treatment for mental manifestations in Hungtington are made mainly on clinical assessment and professional advice because there is little data in this area. Nonpharmacological therapies such as behavioral therapy or interpersonal psychotherapy can be used to treat mental illness, anxiousness, panic disorder, and impatience. Nevertheless, such methods may be constrained in the presence of cognitive problems. In addition to mirtazapine and venlafaxine, which have serotonergic and noradrenergic actions, pharmacological therapies comprise selective serotonin uptake inhibitors. Aggressiveness and insanity are two conditions that can be helped by psychoactive medication. Several drugs have been tried to treat indifference, but no randomized control trials have been carried out [36].

## Conclusions

Over the past 20 years, there has been a significant improvement in both illness awareness and patient treatment. One often overlooks the many years spent in the at-risk and developmental phases, also known as the premanifest phase, prior to the manifestation of symptoms because the typical disease course is even more than 17 years. Both the sufferer as well as the household are affected by HD for the rest of their lives. Articles have multiplied dramatically. Then what's the present viewpoint? Both pathogeneses, as well as the hunt for biomarkers, are the primary subjects of the fundamental investigations. New drug development disrupts the diseased mechanism and will undoubtedly result from a more profound knowledge of pathophysiology. The search is on for medications that can slow down, postpone, or halt the disease's onset. The second problem is the ongoing quest for accurate, easily identifiable, and clinically significant indicators indicating the beginning of the disease's terminal phase. The database research, which is the European Huntington Disease Network's flagship initiative, also intends to set the stage for more considerable investigations whenever medications are made accessible for testing on humans. The finest care for all sufferers and at-risk individuals at this moment is being sought after in tandem with the excellent road to identify treatments for this condition. The discoveries are encouraging; however, there is no doubt that there is a long way to go before a solution can be found.
